# Health Risk Assessment for Exposure to Benzene in Petroleum Refinery Environments

**DOI:** 10.3390/ijerph120100595

**Published:** 2015-01-12

**Authors:** Benjamin Edokpolo, Qiming Jimmy Yu, Des Connell

**Affiliations:** 1 Griffith School of Engineering, Griffith University, Nathan Campus, Brisbane, QLD 4111, Australia; E-Mail: jimmy.yu@griffith.edu.au; 2 Griffith School of Environment, Griffith University, Nathan Campus, Brisbane, QLD 4111, Australia; E-Mail: d.connell@griffith.edu.au

**Keywords:** exposure assessment, health risk assessment, hazard quotient, cancer risk, overall risk probability, petroleum refinery

## Abstract

The health risk resulting from benzene exposure in petroleum refineries was calculated using data from the scientific literature from various countries throughout the world. The exposure data was collated into four scenarios from petroleum refinery environments and plotted as cumulative probability distributions (CPD) plots. Health risk was evaluated for each scenario using the Hazard Quotient (HQ) at 50% (C_EXP50_) and 95% (C_EXP95_) exposure levels. Benzene levels were estimated to pose a significant risk with HQ_50_ > 1 and HQ_95_ > 1 for workers exposed to benzene as base estimates for petroleum refinery workers (Scenario 1), petroleum refinery workers evaluated with personal samplers in Bulgarian refineries (Scenario 2B) and evaluated using air inside petroleum refineries in Bulgarian refineries (Scenario 3B). HQ_50_ < 1 were calculated for petroleum refinery workers with personal samplers in Italian refineries (Scenario 2A), air inside petroleum refineries (Scenario 3A) and air outside petroleum refineries (Scenario 4) in India and Taiwan indicating little possible adverse health effects. Also, HQ_95_ was < 1 for Scenario 4 however potential risk was evaluated for Scenarios 2A and 3A with HQ_95_ > 1. The excess Cancer risk (CR) for lifetime exposure to benzene for all the scenarios was evaluated using the Slope Factor and Overall Risk Probability (ORP) methods. The result suggests a potential cancer risk for exposure to benzene in all the scenarios. However, there is a higher cancer risk at 95% (C_EXP95_) for petroleum refinery workers (2B) with a CR of 48,000 per 10^6^ and exposure to benzene in air inside petroleum refineries (3B) with a CR of 28,000 per 10^6^.

## 1. Introduction

Petroleum refineries and petrochemical plants are major sources of Volatile Aromatic Hydrocarbons (VAHs) in the environment [1]. Benzene is a major VAH emitted during petroleum refinery operations [2] and has been widely used as a solvent in industries such as printing and the manufacture of shoes [3]. It is a confirmed human carcinogen [4] and epidemiological studies have shown it causes the occurrence of acute and chronic leukemia, even at low concentrations [5]. Acute exposure to high benzene concentrations can also affect the central nervous system and cause dizziness, headaches and nausea, while chronic exposure can give rise to more serious adverse health effects such as blood disease, haematotoxicity, genotoxicity, increased levels of persistent chromosome aberrations, reproductive effects and mortality [6,7,8].

Where possible, the use of benzene in manufacturing processes has been reduced by replacement with less hazardous compounds. Hence, benzene is now generally regarded as almost exclusively a product of petroleum refining [9]. Workers in petroleum refineries, including those involved in loading and transportation of petroleum products, may have some level of exposure to benzene [10].

Occupational exposure limits (OELs) have been introduced by various organizations for the management of benzene exposure. As reported in [11] benzene concentrations in modern refineries in Italy have been reported to be less than 3 mg/m^3^, but investigations of petroleum refinery workers in Bulgaria found benzene concentrations levels higher than 3 mg/m^3^ [2]. Exposure to benzene for petroleum refinery workers has been studied in several different countries and a significant body of exposure data is available in the scientific literature. However health risk assessments for benzene other than comparisons with guidelines are rare in the scientific literature.

Health risk assessment for exposure to toxic pollutants is usually carried out to evaluate the adverse effects using single data points to quantify the risk. However, risk assessment using probabilistic techniques utilizes probability distributions to estimate the risk thereby giving an evaluation of variability [12,13,14,15]. This technique gives a quantitative description of uncertainty and variability in evaluating the risk of adverse health effects [16]. In previous work [17,18] we have developed novel techniques for the characterization of human health risk assessment using probabilistic techniques. Previously we have used probabilistic techniques to evaluate the health effects of volatile aromatic hydrocarbons, benzene, toluene and xylene on workers and customers in service stations serving petrol fuel for motor vehicles. This allowed us to overview international data indicating that service station attendants and mechanics repairing dispensing petrol pumps had a significant health risk due to exposure to benzene.

The aim of this study was to collect and collate exposure data for benzene in petroleum refineries environment on a global scale and conduct a risk assessment to evaluate the possible adverse health effects.

## 2. Methodology

### 2.1. Research Strategy

The strategy used in this research involved collection and collation of benzene exposure data and guideline values from the scientific literature. Like data sets presented in Table 1, were combined together and then divided into Scenarios according to location and setting. The data sets for each Scenario were used to develop Cumulative Probability Distribution (CPD) plots. From the CPD plots, the Hazard Quotient and Cancer Risk were evaluated at 50% (C_EXP50_) and 95% (C_EXP95_) cumulative probability of benzene exposure levels. The C_EXP50_ level gave an evaluation relevant to most of the exposed population while C_EXP95_ was relevant to the 5% most exposed group. On the other hand, Overall Risk Probability was an estimated value relative to the whole population.

**Table 1:** Investigations of benzene concentrations in petroleum refineries.

Reference	Description	Country	Sampling Method
[11]	Cytogenic biomonitoring on a group of petroleum refinery workers	Italy (two different Italian petroleum refineries)	P ^*^
[2]	Cytogenic effects of Bulgarian petroleum refinery workers chronically exposed to benzene	Bulgaria (NEFTOCHIM oil company in Burgas)	P ^*^ and S ^*^
[19]	Retrospective exposure assessment for benzene in the Australian petroleum industry	Australia (nine companies with employees participating in health watch)	BE ^*^
[20]	Ensuring comparability of benzene exposure estimates across three nested case-control studies in the petroleum industry in support of a pooled epidemiological analysis	Canada, Australia, United Kingdom	BE ^*^
[21]	Retrospective estimation of exposure to benzene in a leukaemia case-control study of petroleum marketing and distribution workers in the United Kingdom	United Kingdom (four companies in the petroleum marketing and distribution industry in the UK)	BE ^*^
[1]	Seasonal variation of toxic benzene emissions in petroleum refinery	India (Digboi petroleum refinery at Gowahati	S ^*^
[22]	Monitoring and analysis of volatile organic compounds around an oil refinery	Italy (a petroleum refinery in Valle Galeria, Rome)	S ^*^
[10]	Volatile organic compounds in ambient air of Kaohsiung petroleum refinery	Taiwan, Kaohsiung refinery	S ^*^

^*^ P, personal sampling; S, static sampling; BE, base estimate.

#### 2.1.1. Data Collection

Data sets for benzene exposure in petroleum refinery environments used in this study were gathered from the scientific literature using various search engines such as Google, Web of Knowledge, PubMed, Toxnet, Medline and Science Direct. Each reference provided one or more sets of benzene measurements, with each set representing measurements for a sampling location, activity or occupation (Table 1) [1,2,10,11,19,20,21,23].

#### 2.1.2. Criteria for Data Selection

The health risk was focused on evaluating exposure data on benzene concentrations in the ambient air of petroleum refineries. Only data sets reported as individual concentrations and base estimates concentrations were utilized for consistency since a number of data sets were reported as mean concentrations. These data sets (mean data) were not included in the risk assessment analysis since they cannot be combined and interpreted with the datasets on individual measurements and base estimates.

#### 2.1.3. Preparation of Probability Distribution Plots

The data sets for benzene exposure were used to develop Cumulative Probability Distribution (CPD) plots by using Microsoft Excel. CP (%) was calculated from Equation (1):

(1) CP(%) = (i/n+1) ×100

where CP is cumulative probability (%); *i*, *i*th point; n, total number of data points. The linear regression equations of the CPD plots were usually calculated between approximately 10%–90% of the Cumulative Probability distribution since this represents the approximately linear part of the CPD plots when a normal distribution occurs.

#### 2.1.4. Guideline Values for Benzene

The exposure limits for occupational exposure to benzene from various organizations such as European Commission, OSHA, NIOSH, ACIGH and SWA and Air Quality Guidelines (AQGs) and United Kingdom are summarized in Table 2 [22,24,25,26,27]. Exposure evaluation of benzene concentrations in the various scenarios were for occupational and general population exposure. Occupational exposure limits were used to compare exposures to benzene for Scenarios 1, 2A, 2B, 3A and 3B (occupational exposure), while Air Quality Guideline (AQGs) were used to compare exposure to benzene in Scenario 4 (exposure to people external to the petroleum refineries).

### 2.2. Data Analysis

#### 2.2.1. Background

The data sets were obtained from the publications listed in Table 1. The benzene concentration data were converted from mg/m^3^, ppm and ppb to a uniform unit of µg/m^3^. The data sets that were used to develop CPD plots for exposure to benzene were categorized into Scenarios as outlined below.

#### 2.2.2. Scenario 1—Exposure to Benzene as Base Estimate Concentrations for Petroleum Refinery Workers

This scenario represents benzene concentrations collected as base estimate concentrations for retrospective benzene exposures in petroleum industries from studies using similar methods in deriving the base estimates from benzene measurements. The studies were for early 1940 to 1996 for the Australian study [19]; 1909 to 1989 for the Canadian study [20]; 1902 to 1992 for the United Kingdom study [21]. Base estimates were calculated for available measurements of benzene concentrations during these periods. However, in situations were measured benzene data was not available, benzene exposure was estimated to derive the base estimate. The data sets used in this Scenario were obtained from [19,20,21].

**Table 2:** Standards and guidelines for exposure to benzene.

Regulatory Body	Description	Benzene Concentration (µg/m^3^)
Occupational Exposure Limits (OEL)		
American Conference of Governmental Industrial Hygienists (ACGIH), USA	Threshold Limit Values (TLV)	1600
Occupational Safety and Health Administration (OSHA), USA	Permissible Exposure Limit (PEL)	3250
National Institute for Occupational Safety and Health (NIOSH), USA	Recommended Exposure Limit (REL)	325
Safe Work Australia (SWA)	Occupational Exposure limit (OEL)	3250
European Directives 2000/39/EC and 97/42/EC (ED)	Limit Value (LV)	3250
Air Quality Guidelines (AQGs)		
European Union Directives 2000/69/EC	Annual mean	5
Expert Panel on Air Quality Standards (EPAQS), United Kingdom	Annual mean	16.25

#### 2.2.3. Scenario 2—Exposure to Benzene for Petroleum Refinery Workers

This scenario was for petroleum refinery workers in different occupation within the petroleum refineries exposed to benzene. The concentrations of benzene in air were collected by the workers wearing personal air sampling pumps. The data sets used in this scenario were obtained from [2,11].

#### 2.2.4. Scenario 3—Benzene Concentrations in Air Inside the Petroleum Refineries

The data sets were derived from air samples of benzene taken within various work locations inside the petroleum refineries. Measurements of benzene concentration levels were obtained by using air sampling pumps positioned at various locations inside the petroleum refineries. The data sets used in this scenario were obtained from [1,2,10].

#### 2.2.5. Scenario 4—Benzene Concentrations in Air Outside the Petroleum Refineries

The data sets obtained for this scenario were for emissions of benzene from petroleum refineries to the immediate surroundings giving exposure to people living near the petroleum refineries. Benzene concentrations were obtained around the petroleum refineries at a maximum distance of 2 km by using air sampling pumps at different sampling locations near the petroleum refineries. The data sets used in this scenario were obtained from [1,10,22].

### 2.3. Risk Characterization

#### 2.3.1. Exposure Calculation

**Figure 1:**
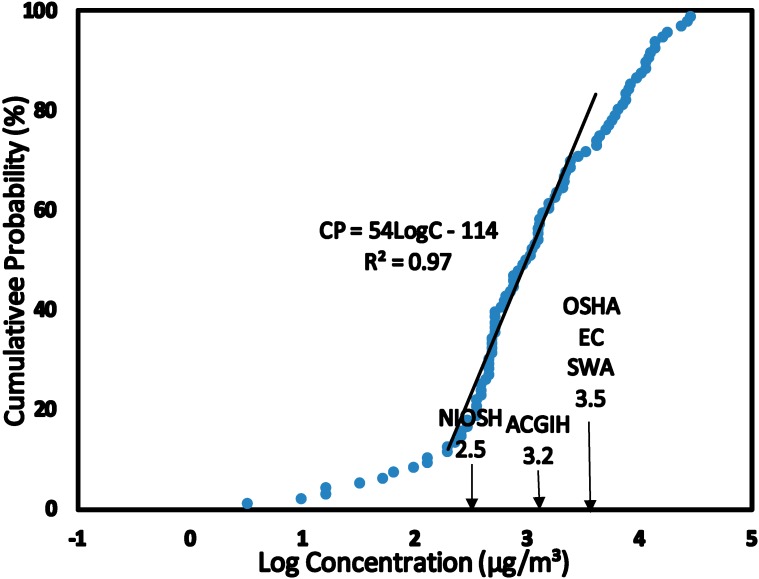
SCENARIO 1—Exposure to Benzene as Base Estimates for Petroleum Refinery Workers. Retrospective exposure to benzene concentrations as base estimate concentrations for petroleum refinery workers in Australia (1940 to 1989), Canada (1902 to 1996) and United Kingdom (1906 to 1989). Base estimate concentration data for years prior to 1970 is based on modelling and is included in this plot.

**Figure 2:**
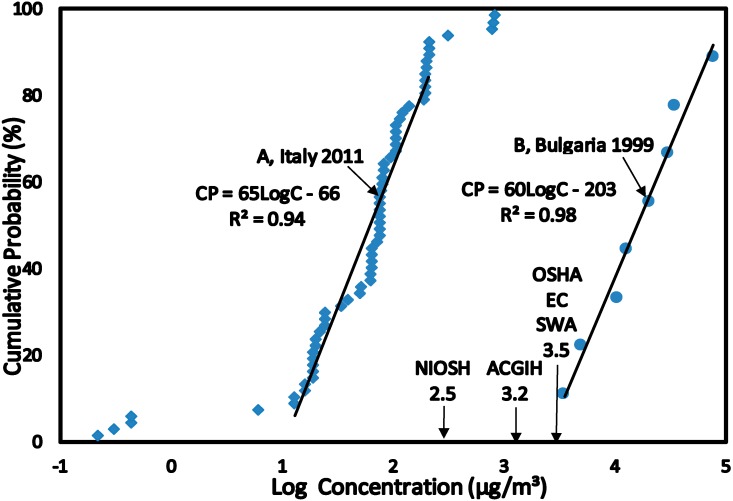
SCENARIO 2—Exposure to Benzene for Petroleum Refinery Workers. Exposure to benzene concentrations in air measured using personal sampling techniques for petroleum refinery workers in Italy (**A**) 2011 and Bulgaria (**B**) 1999.

**Figure 3:**
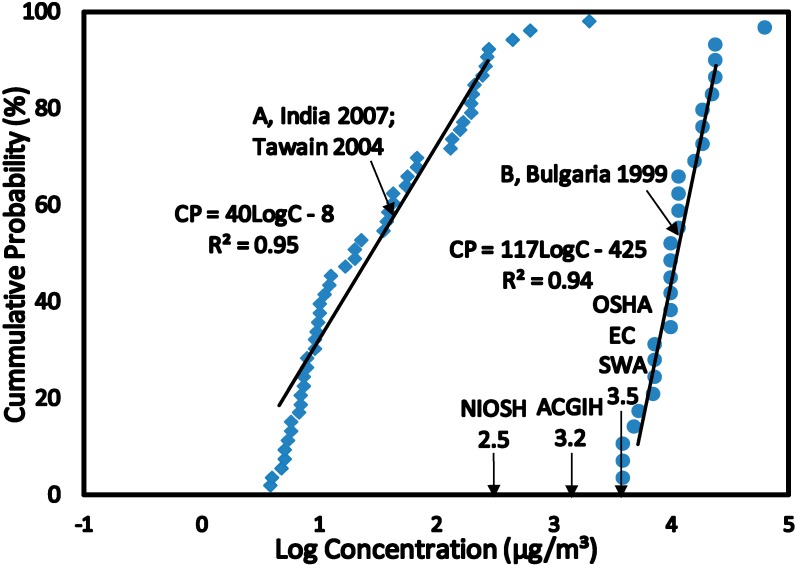
SCENARIO 3—Benzene Concentrations in Air inside Petroleum Refineries. Benzene concentrations in air measured by static sampling techniques inside petroleum refineries in India (**A**) 2007 and Taiwan (A) 2004 and in Bulgaria (**B**) 1999.

**Figure 4:**
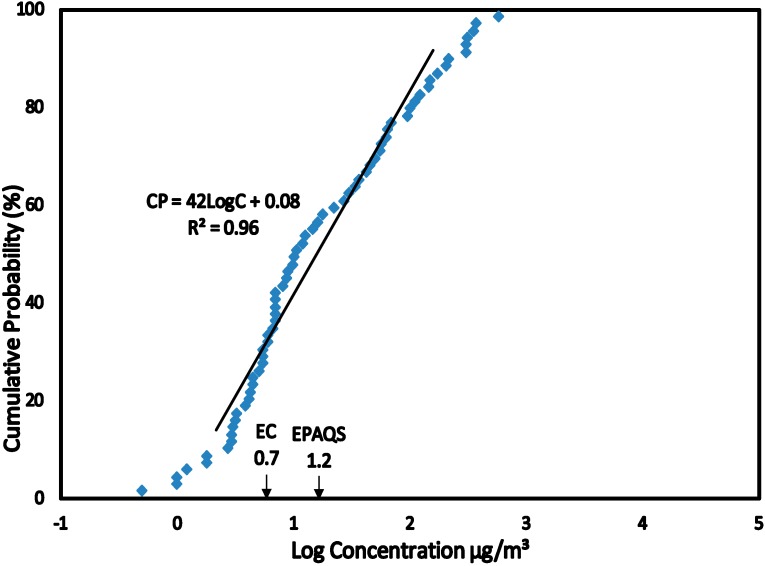
SCENARIO 4—Benzene Concentrations in Air outside Petroleum Refineries. Benzene concentrations in air measured by static sampling techniques around the petroleum refineries in India (2000), Italy (2004) and Taiwan (2008) at a maximum distance of 2 km.

The data sets for exposure to benzene were categorized into related Scenarios 1 to 4 (see Section 3.1) and converted into CPD plots (see Figure 1, Figure 2, Figure 3 and Figure 4). This allowed the estimation of the C_EXP50_ (the median level which represents the main group) and C_EXP95_ representing the highest exposed group in the population levels of exposure of the population within each scenario. The benzene concentrations (Scenario 1 to 4) were converted from µg/m^3^ to µg/kg/day in terms of Lifetime Average Daily Dose (LADD) using values summarized in Table 3. The LADD were used in calculating the Hazard Quotient, Cancer Risk and Overall Risk Probability. The values of USEPA Inhalation Reference Dose (RfD) and Slope Factor (SF) for estimating the HQ and CR were summarized in Table 3 [28,29,30].

The Lifetime Average Daily Doses (LADD) (µg/kg/day) for exposure to benzene concentrations were calculated for all Scenarios using the default values in Table 3 with Equation (2):

(2) $$LADD=\;[C_{EXP}\;\times IR\;\times EL\times \;ED]/[BW\;\times LT]$$

where C*_EXP_* is exposure concentration (µg/m^3^); IR, Inhalation Rate (m^3^/day); EL, Exposure Length (day/day); ED, the Exposure Duration (days); BW, Body Weight (kg); LT, Lifetime (days).

**Table 3:** Summary of default exposure factors.

Parameter	Unit	Default Value
Lifetime (LT)	years	70
Body Weight (BW)	kg	70
Exposure Length (EL)	day/day	0.33 (8 h/day) (workers)
		0.17 (4 h/day) (outdoor)
Exposure Duration (ED)	years	25 (commercial/industrial)
		30 (residential)
Inhalation Rate (IR)	m^3^/day	20
Inhalation Reference Dose (RfD)	mg/kg/day	0.0085
Slope Factor	(mg/kg/day)^−1^	0.0273

LT = 7 days/week × 52 weeks/year × 70 years = 25,480 days (Scenario 1 to 4); ED = 5 days/week × 48 weeks/year × 25 years = 6000 days (Scenario 1and 2); ED = 7 days/week × 52 weeks/year × 25 years = 9100 days (Scenario 3); ED = 7 days/week × 52 weeks/year × 30 years = 10,920 days (Scenario 4); EL = 0.33 day/day (8 h/day) (Scenarios 1, 2 and 3); EL = 0.17 day/day (4 h/day) (Scenario 4).

#### 2.3.2. Use of the Hazard Quotient (HQ)

The HQ method of risk characterization was used to estimate the adverse health effects for exposure to benzene. The USEPA Reference Dose (RfD) derived for benzene was used to estimate the HQ for all Scenarios by using Equation (3). Benzene exposures were estimated at the median level (C_EXP50_) which represents the main group of individuals and the 95% level (C_EXP95_) representing the highest exposed group in the population. This highly exposed group occurs at a level of 5% in the population and the median group represents over 50% in the population. Benzene concentrations at C_EXP50_ and C_EXP95_ were obtained from the CPD plots (Figure 1, Figure 2, Figure 3 and Figure 4) and converted to LADD using Equation (2). HQ was estimated at C_EXP50_ and C_EXP95_ using Equation (3):

(3) HQ=LADD/RfD

where HQ is the Hazard Quotient; LADD, lifetime average daily dose (µg/kg/day); RfD, USEPA reference dose (µg/kg/day) (Table 3).

#### 2.3.3. Cancer Risk

Cancer risk is expressed as excess risk of developing cancer over a lifetime of exposure (70 years).

The USEPA inhalation slope factor derived for benzene was used to quantitatively estimate the excess cancer risk at C_EXP50_ and C_EXP95_ in terms of lifetime exposure (LADD) in the various scenarios by using Equation (4):

Cancer Risk = LADD (µg/kg/day) × SF (µg/kg/day)^−1^ (4)

where SF is slope factor for benzene (Table 3).

#### 2.3.4. Evaluation Using Overall Risk Probability (ORP)

**Figure 5:**
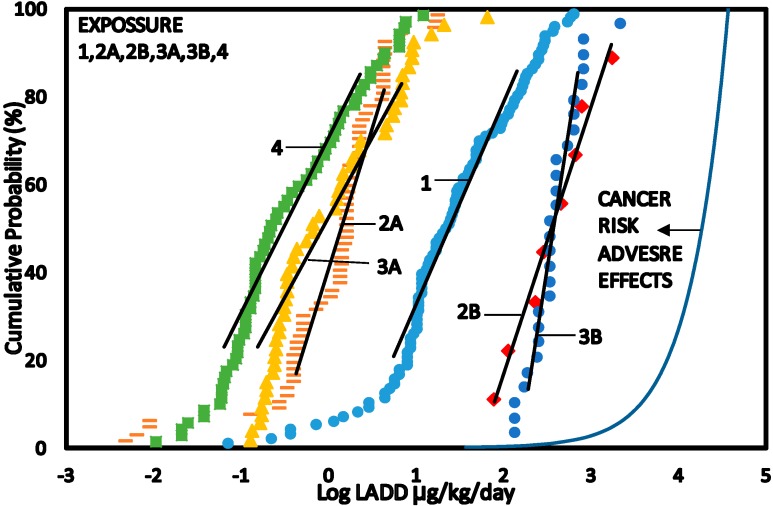
CPD plots of exposure to benzene as Lifetime Average Daily Dose (LADD) for Scenario 1–4 and cancer risk adverse effects dose—Response relationship.

The ORP method is based on the use of Overall Risk Probability (ORP) curve. The ORP curve is the plot of exposure exceedence values (1—CP) against the corresponding CP values for dose-adverse effects (Figure 5). 

A detailed description of overall risk probability in risk assessment has been discussed in [16]. The CPD plots for benzene exposure were derived from the data sets for benzene concentrations that were converted to Life Time Average Daily Dose (LADD) and the calculated cancer risk adverse effect relationship obtained using Equation (4) (Figure 5).

## 3. Results and Discussion

### 3.1. Scenario 1—Exposure to Benzene as Base Estimates for Petroleum Refinery Workers

The CPD plot as shown in Figure 1 is for exposure to benzene concentrations for refinery workers in Australia, Canada and United Kingdom from 1902 to 1996. The linear equation has a slope of 54 and correlation coefficient (*R*^2^) of 0.97 indicating a normal distribution. At C_EXP50_, exposure to benzene was higher than NIOSH REL but lower than ACIGH TLV, OSHA PEL, EC LV and SWA OEL. However, at C_EXP95_ exposure to benzene was higher than NIOSH REL, ACIGH TLV, OSHA PEL, EC LV and SWA OEL. The workers in the highly exposed group reported by the high exposure concentrations in the CPD plots were workers involved in activities such as drum fillers, large terminal operator, gauging, line pigging, rail car loading, refueling, tanker loading and cleaning.

### 3.2. Scenario 2—Exposure to Benzene for Petroleum Refinery Workers

The CPD plots (Figure 2) are for exposure to benzene concentrations for petroleum refinery workers in Italy, 2011 (A) and Bulgaria, 1999 (B). The linear regression equations had correlation coefficients (*R*^2^) > 0.94 for both CPD plots indicating normal distributions. The CPD plots of Scenario 2A and 2B have almost identical slopes of 65 and 60, respectively. This implies that there was a comparatively wide range of benzene concentration distribution. At C_EXP50_ and C_EXP95_ exposure to benzene for Scenario 2A was lower than NIOSH REL, ACIGH TLV, OSHA PEL, EC LV and SWA OEL. While at C_EXP50_ and C_EXP95_ exposure to benzene in Scenario 2B was higher than NIOSH REL, ACIGH TLV, OSHA PEL, EC LV and SWA OEL. The high exposure to benzene was for workers in transport and storage of petroleum products facility, benzene manufacturing plant and ethylbenzene—styrene manufacturing plant.

### 3.3. Scenario 3—Benzene Concentrations in Air Inside the Petroleum Refineries

The CPD plots in Figure 3 is for benzene concentrations in the air for petroleum refinery in India (A), Taiwan (A) and Bulgaria (B). The linear regression equations had correlation coefficients (*R*^2^) > 0.94 for both CPD plots indicating high level of linearity in the distributions. The slope for Scenario 3C is 40 while Scenario 3A has a slope of 117, indicating a relatively wide range of benzene concentration distribution for both CPD plots. At C_EXP50_ and C_EXP95_ exposure to benzene for Scenario 3A was lower than NIOSH REL, ACIGH TLV, OSHA PEL, EC LV and SWA OEL. While at C_EXP50_ and C_EXP95_ exposure to benzene in Scenario 3B was higher than NIOSH REL, ACIGH TLV, OSHA PEL, EC LV and SWA OEL. The high exposure to benzene was for workers in transport and storage of petroleum products facility, benzene manufacturing plant and ethylbenzene—styrene manufacturing plant.

### 3.4. Scenario 4—Benzene Concentrations in Air Outside the Petroleum Refineries

The concentrations of benzene in the air measured outside the petroleum refineries in India, Italy and Taiwan were plotted as CPD plots (Figure 4). The linear regression equations had a correlation coefficient (*R*^2^) > 0.96 indicating a high level of linearity in the distribution and a normal distribution. The slope for CPD plot (Figure 4) was 42 indicating a relatively wide range of benzene concentrations. The exposure to benzene outside the refineries was compared to the Air Quality Guidelines (AQG) presented in Table 2. At C_EXP50_ benzene concentration levels were higher than AQG for European Commission but lower than AQG for United Kingdom and at C_EXP95_ indicated that exposures to benzene were higher than AQG for European Commission but lower than AQG for United Kingdom (Figure 4).

### 3.5. Risk Characterization

#### 3.5.1. Hazard Quotient (HQ)

The calculated LADD at C_EXP50_ and C_EXP95_ for exposure to benzene was used in estimating the HQ (Equation (3)) and the results were summarized in Table 4. HQ_50_ were < 1 for petroleum refinery workers (Scenario 2A), benzene concentrations in air inside the petroleum refineries (Scenario 3A), and benzene concentrations in air outside the petroleum refineries (Scenario 4). This result suggests minimal risk to the majority of the population in these exposure Scenarios (2A, 3A and 4). Also, HQ_95_ was <1 for Scenario 4 suggesting minimal risk to the high exposed group. However, HQ_95_ were >1 for Scenarios 2A and 3A indicating possible risk to adverse effects. HQ_50_ and HQ_95_ for lifetime exposure to benzene for Scenario 1 (base estimates for petroleum refinery workers), 2B (exposure to benzene for petroleum refinery workers) and 3B (benzene concentrations in air inside the petroleum refineries) were >1 indicating possible adverse health effects for the main group of exposed individuals and the high exposed.

#### 3.5.2. Cancer Risk Calculated

The excess CR was calculated for exposure to benzene at the median level (C_EXP50_) which represents the main group of exposed individuals and the 95% level (C_EXP95_) representing the highest exposed group in the population (Scenarios 1 to 4) and the results were presented in Table 4. The results suggest different levels of cancer risk for chronic exposure to benzene for the main group and highest exposed group in the various Scenarios. At C_EXP50_, the excess cancer risk in terms of lifetime exposure to benzene for Scenario 2A (petroleum refinery workers), 3A (benzene concentrations in air inside the petroleum refineries) and 4 (benzene concentrations in air outside the petroleum refineries) are very low in the range of 6 to 18 per 10^6^ as compared to Scenario 1 (base estimates for petroleum refinery workers), 2B (petroleum refinery workers) and 3B (benzene concentrations in air inside the petroleum refineries) that is in the range of 590 to 10,000 per 10^6^. On the other hand, at C_EXP95_, the highly exposed group that occurs at a level of 5% in the population, the excess cancer risk in terms of lifetime exposure to benzene for Scenario 2A (petroleum refinery workers), 3A (benzene concentrations in air inside the petroleum refineries) and 4 (benzene concentrations in air outside the petroleum refineries) are very low in the range of 200 to 460 per 10^6^ as compared to Scenario 1 (base estimates for petroleum refinery workers), 2B (petroleum refinery workers) and 3B (benzene concentrations in air inside the petroleum refineries) that is in the range of 10,000 to 48,000 per 10^6^. The cancer risk estimated at C_EXP95_ is only for 5% of the exposed population. The significance difference in the cancer risk estimated is as a result of higher concentration levels of benzene observed in 2B (petroleum refinery workers) and 3B (benzene concentrations in air inside the petroleum refineries) that was 17 to 1400 and 4.6 to 230 times higher than Scenarios 1 to 4 at (C_EXP50_) and (C_EXP95_) respectively.

#### 3.5.3. Overall Risk Probability (ORP)

**Figure 6:**
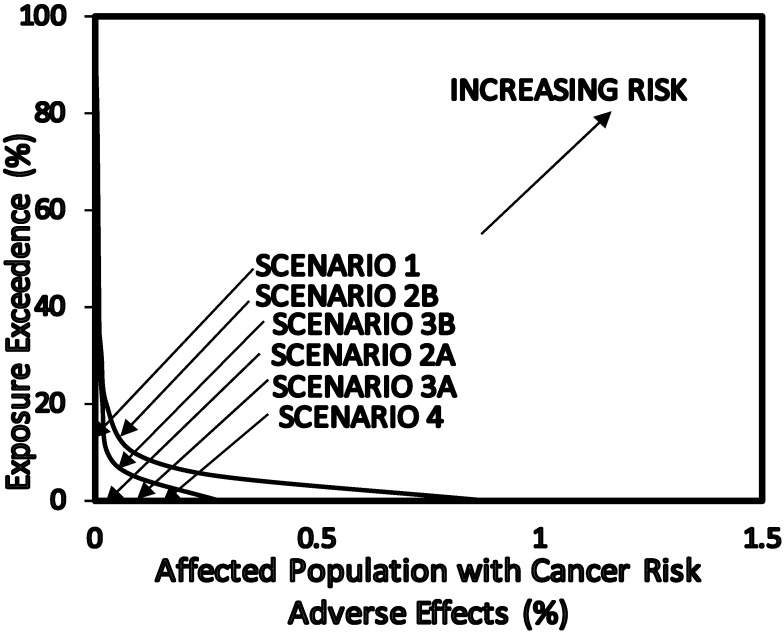
Overall risk probability (ORP) for cancer risk as a result of exposure to benzene concentrations in petroleum refinery environments.

Cancer risk adverse dose—response relationship are shown in Figure 5. The ORP plots are shown in Figure 6.The area under the ORP curves were calculated to obtain values of Overall Risk Probability (Table 4). ORP of 0.17% (1700 per 10^6^) was obtained for Scenario 1 (base estimates for petroleum refinery workers), 0.011% (110 per 10^6^) for Scenario 2A (petroleum refinery workers), 4.8% (48,000 per 10^6^) for Scenario 2B (exposure to benzene for petroleum refinery workers), 0.015% (150 per 10^6^) for Scenario 3A (benzene concentrations in air inside the petroleum refineries), 1.7% (17,000 per 10^6^) for Scenario 3B (benzene concentrations in air inside the petroleum refineries) and 0.009% (110 per 10^6^) for Scenario 4 (benzene concentrations in air outside the petroleum refineries).

Overall the ORP and the CR are in reasonable agreement (Table 4). The difference between the ORP method and CR method is that the CR were calculated for the highly exposed group (C_EXP95_) and the main group of individuals (C_EXP50_) in the population, while with ORP all of the exposed population were taken into consideration as shown in Table 4.

**Table 4:** Health risk characterization for exposure to benzene.

Scenario	C_EXP50_	LADD_50 _(µg/kg/Day)	C_EXP95_	LADD_95_ (µg/kg/Day)	HQ at LADD_50_	HQ at LADD_95_	CR per 10^6^ at LADD_50_	CR per 10^6^ at LADD_95_	CR per 10^6^ Estimated by ORP
Scenario 1 (Base Estimate)	980	22	17,000	380	2.5	44	590	10,000	1700
Scenario 2A (Refinery workers)	75	1.7	690	16	0.20	1.9	45	420	110
Scenario 2B (Refinery workers)	17,000	370	79,000	1800	43	210	10,000	48,000	44,000
Scenario 3A (Inside Refinery)	22	0.6	480	14	0.068	1.8	18	460	150
Scenario 3B (Inside Refinery)	11,000	340	28,000	1000	39	120	9200	28,000	17,000
Scenario 4 (Outside refinery)	12	0.21	350	7.3	0.024	0.85	6.0	200	110

## 4. Conclusions

Benzene levels were estimated to pose a significant risk with HQ_50_ > 1 and HQ_95_ > 1 for workers exposed to benzene as base estimates for petroleum refinery workers (Scenario 1), petroleum refinery workers evaluated with personal samplers in Bulgarian refineries (2B) and evaluated using air concentrations inside petroleum refineries in Bulgarian refineries (3B). On the other hand HQ_50_ were <1 for lifetime exposure to benzene in petroleum refinery workers (Scenario 2A), benzene concentrations in air inside the petroleum refineries (Scenario 3A), and benzene concentrations in air outside the petroleum refineries (Scenario 4) suggesting minimal risk to the majority of the population in these exposure Scenarios. HQ_95_ was <1 for Scenario 4 suggesting minimal risk to the high exposed group however, HQ_95_ were >1 for Scenarios 2A and 3A indicating possible risk to human health for the high exposed group. The excess cancer risk for lifetime exposure to benzene for all the Scenarios was evaluated using the Slope Factor method at C_EXP50_ and C_EXP95_ and also using the ORP method. The two methods showed a reasonable level of agreement. With the ORP method, workers in petroleum refineries in Scenario 2B were observed to have the highest cancer risk 44,000 per 10^6^ followed by those evaluated with data from air inside the petroleum refineries in Scenario 3B with cancer risk of 17,000 per 10^6^ and base estimates for petroleum refinery workers Scenario 1 with cancer risk of 1700 per 10^6^.
